# Clinical Feasibility of a High-Resolution Thermal Monitoring Sheet for Superficial Hyperthermia in Breast Cancer Patients

**DOI:** 10.3390/cancers12123644

**Published:** 2020-12-04

**Authors:** Akke Bakker, Remko Zweije, Henny Petra Kok, Merel Willemijn Kolff, H. J. G. Desiree van den Bongard, Manfred Schmidt, Geertjan van Tienhoven, Hans Crezee

**Affiliations:** 1 Department of Radiation Oncology, Amsterdam UMC, 1105 AZ Amsterdam, The Netherlands; r.zweije@amsterdamumc.nl (R.Z.); h.p.kok@amsterdamumc.nl (H.P.K.); m.w.kolff@amsterdamumc.nl (M.W.K.); h.j.vandenbongard@amsterdamumc.nl (H.J.G.D.v.d.B.); g.vantienhoven@amsterdamumc.nl (G.v.T.); h.crezee@amsterdamumc.nl (H.C.); 2 Department of Radiation Oncology, University Hospital Erlangen, 91054 Erlangen, Germany; manfred.schmidt@uk-erlangen.de

**Keywords:** hyperthermia, radiation oncology, thermal monitoring, quality assurance

## Abstract

**Simple Summary:**

Hyperthermia, i.e., heating tumors to 41–43 °C, combined with radiotherapy improves treatment response, for patients with recurrent breast cancer after previous irradiation. During hyperthermia of superficial tumors, the skin surface temperature must be monitored to ensure that therapeutic temperatures are reached without hotspots that can cause additional toxicity. A thin sheet with a dense grid of 56 temperature sensors was developed, this sheet is placed on the skin of the patient. The influence of the sheet on the hyperthermia applicator performance was investigated and found to be negligible. Next, the clinical feasibility was evaluated in 10 women with locoregional recurrent breast cancer, and resulted in precise monitoring of skin surface temperatures. In conclusion, this novel method can be implemented for thermal monitoring of the skin surface to ensure treatment quality during superficial hyperthermia treatment of patients with locoregional recurrent breast cancer.

**Abstract:**

*Background:* Accurate monitoring of skin surface temperatures is necessary to ensure treatment quality during superficial hyperthermia. A high-resolution thermal monitoring sheet (TMS) was developed to monitor the skin surface temperature distribution. The influence of the TMS on applicator performance was investigated, feasibility and ability to reliably monitor the temperature distribution were evaluated in a clinical study. *Methods:* Phantom experiments were performed to determine the influence of the TMS on power deposition patterns, applicator efficiency, and heat transfer of the water bolus for 434 and 915 MHz applicators. Clinical feasibility was evaluated in 10 women with locoregional recurrent breast cancer. Skin surface temperatures during consecutive treatments were monitored alternatingly with either standard Amsterdam UMC thermometry or TMS. Treatments were compared using (generalized) linear mixed models. *Results:* The TMS did not significantly affect power deposition patterns and applicator efficiency (1–2%), the reduced heat transfer of the water boluses (51–56%) could be compensated by adjusting the water bolus flow. Skin surface temperatures were monitored reliably, and no alteration of thermal toxicity was observed compared to standard Amsterdam UMC thermometry. *Conclusion:* Clinical application of the TMS is feasible. Power deposition patterns and applicator efficiency were not affected. Surface temperatures were monitored reliably.

## 1. Introduction

Numerous clinical studies have shown the benefit and efficacy of hyperthermia (i.e., heating tumors to 40–43 °C) as a radiotherapy sensitizer in the treatment of locoregional breast cancer in previously irradiated area [1,2,3,4,5]. Heating superficial recurrences is typically achieved with microwave hyperthermia systems, which couple 434 or 915 MHz microwave energy via an external water bolus—a water bag positioned between applicator and skin—into the target volume to induce local heating. Distilled water at temperatures varying from 39 to 43 °C is circulated through the water bolus to actively cool the skin to prevent temperature hotspots on the skin surface that might result in thermal toxicity, this allows for the application of more antenna power and thereby improves the penetration depth of heat [6].

Adequate temperature monitoring during treatment is important to ensure effective heating, since thermal dose effect relationships have been found in both prospective and retrospective studies [3,4]. Temperature monitoring is also important to help avoiding excessive temperatures (>43.5 °C), thereby reducing the risk of thermal toxicity [4,7]. However, temperature monitoring during clinical superficial hyperthermia is generally limited by poor spatial resolution. International quality assurance guidelines advise a minimum number of five sensors on the skin surface [8], and commercial microwave hyperthermia systems until now generally offer eight single temperature sensors for temperature monitoring with their applicators (ALBA ON 4000, Medlogix, Rome, Italy; BSD-500, Pyrexar, Salt Lake City, USA). However, a recent study demonstrated that at least 50 sensors would be necessary for adequate mapping of the skin temperature when using a frequently applied applicator that effectively heats an area of 400 cm^2^ [9].

Current methods to improve spatial thermometry resolution during superficial hyperthermia treatment include thermal mapping, i.e., pulling a limited number of temperature sensors through a fixed trajectory several times during treatment [10], and increasing the number of temperature sensors by purchase and clinical implementation of additional multichannel thermometry systems [9,11]. The latter option may be combined with a thermal monitoring sheet [11,12]. Although these methods substantially improve the spatial resolution of thermal monitoring, they are expensive, time-consuming, increase the treatment burden for the patient, and/or require experienced users, thereby limiting clinical implementation in hyperthermia centers worldwide.

Recently, a high-resolution 56 sensor thermal monitoring sheet (TMS) with a monitoring area of 162.5 × 140 mm, was developed to improve thermal monitoring of the skin surface (Medlogix, Rome, Italy). Temperature monitoring with the TMS was found to be accurate, stable during 2 h and had a high temperature resolution. The TMS could follow body contours and was compatible with 434 and 915 MHz electromagnetic fields. [4] Before the TMS can be used in clinical practice, the influence of the TMS on applicator performance and skin temperature distribution should be evaluated. 

Quality assurance (QA) guidelines have been developed by the European Society for Hyperthermic Oncology (ESHO) to characterize applicator performance, thereby ensuring treatment quality [13]. The main characteristics determining applicator performance that can be affected by the TMS are the effective field size (EFS) and the efficiency. The EFS is defined by the area covered by 50% of maximum specific absorption rate (SAR) contour in the 10 mm deep plane under the applicator [13]. In addition, the heat transfer of the water bolus to the skin surface may be affected by the TMS. 

The aim of this paper is to evaluate the effect of application of the TMS on treatment delivery regarding applicator performance and temperature monitoring of the skin surface during superficial hyperthermia. To this end we evaluated applicator efficiency, power deposition patterns, including EFS, and water bolus heat transfer in phantom experiments for 434 and 915 MHz applicators. Next, temperature distributions were evaluated and compared with standard of care thermometry in 10 patients with locoregional recurrent breast cancer in a previously irradiated area. 

## 2. Materials and Methods

### 2.1. Amsterdam UMC Standard of Care Thermometry

**Figure 1:**
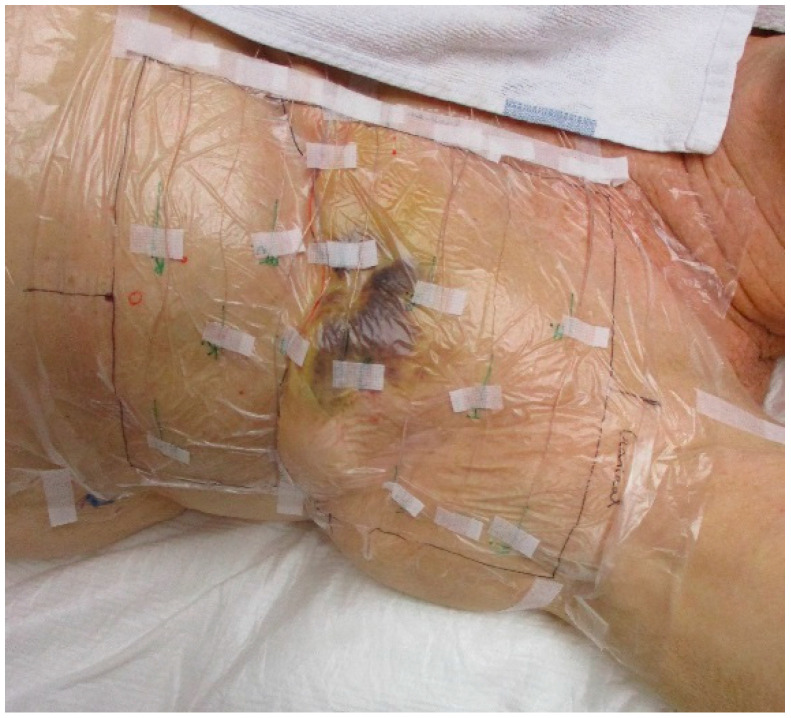
The standard of care thermometry during hyperthermia treatments at the Amsterdam University Medical Centers. Multiple seven-point thermocouples probes are distributed over the skin surface of the target area. To allow accurate positioning and for hygienic reasons a 10 µm high density polyethylene plastic foil is positioned on the chest wall of the patient and the thermocouple probes are placed on the foil.

Standard of care thermometry during superficial hyperthermia at Amsterdam UMC consists of seven-point copper-constantan thermocouple probes (Volenec RD Int., Hradec Králové, Czech Republic) connected to a 196-channel thermometry system (UMCU, Utrecht, the Netherlands) [14]. Typically, one to three probes are placed invasively (if possible), two to six probes on scar tissue, and six to 15 seven-point thermocouple probes are evenly distributed over the target area. A 10 µm high density polyethylene plastic foil is positioned between the skin surface and the thermocouple probes for hygienic reasons and to allow accurate and reproducible probe positioning, the latter is achieved by indicating the intended probe positions on the disposable foil prior to each treatment session (Figure 1). 

### 2.2. Thermal Monitoring Sheet (TMS)

**Figure 2:**
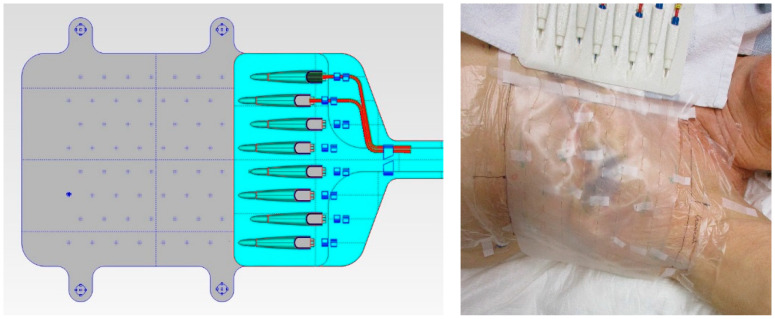
The left picture is a schematic drawing of the thermal monitoring sheet (TMS; Medlogix, Rome, Italy). Dimensions of the transparent silicone sheet are 212.5 × 166 × 0.3 mm (l × w × h) with eight seven-sensor thermocouple probes resulting in a monitoring area of 162.5 × 140 mm. The right picture shows a photograph of the TMS on the chest wall of a study patient.

The TMS was developed by Medlogix (Rome, Italy) in collaboration with Amsterdam University Medical Centers (UMC) to ensure fast, precise, and reproducible sensor placement during superficial hyperthermia treatment and to allow an accurate reconstruction of the skin surface temperature. The TMS consists of a semirigid supportive structure, attached to a flexible silicone sheet with eight multisensor thermocouple probes (Volenec RD Int., Hradec Králové, Czech Republic) laced through the sheet every 20 mm, yielding in total 56 sensors with a 20 × 25 mm spatial resolution (Figure 2). The monitoring area of the TMS is 162.5 × 140 mm. The sensors are positioned on the patient side of the silicone sheet, to ensure optimal thermal contact with the skin surface [15]. The initial version of the TMS had a thickness of 0.5 mm; the final, more flexible version has a thickness of 0.3 mm and is used in this paper for QA evaluation.

### 2.3. Phantom Experiments

Phantom experiments were conducted to evaluate the effect of the TMS on applicator performance. Hyperthermia applicators operating at 434 or 915 MHz are commonly used for heating superficial target areas which are not extending more than 3–4 cm under the skin. A bag with water—referred to as water bolus—is placed between the applicator and skin to transfer the microwave energy into the target area without reflection into the skin. The distilled water in this water bolus is heated to 39–43 °C to ensure that the skin surface temperature is at the therapeutic level. These applicators come in various sizes; an applicator with an effective field size that covers the entire tumor target area was selected. Table 1 gives the specifications of the applicators selected for the phantom experiments and the clinical feasibility study, which represent a typical range of properties, target size, and penetration depth often encountered in clinical practice [16].

The presence of the TMS between the water bolus and the skin surface might affect the magnitude and shape of the power deposition pattern, influencing the effective heating area of the applicators. Therefore, the power deposition patterns and QA characteristics EFS and efficiency will be evaluated. Furthermore, the heat transfer of the water bolus to the skin surface may be reduced when additional layers of material such as the TMS are added between the water bolus and the skin surface [12]. An overview of the experiments and different phantom set-ups used to evaluate applicator efficiency, power deposition patterns, and heat transfer of the water bolus is presented in Table 2.

For the phantom tests we used commercially available applicators operating at the two most commonly used frequencies for superficial hyperthermia, 434 and 915 MHz. Evaluating both frequencies is of interest as the shorter wavelength at 915 MHz could be associated with more pick up of EM radiation by the thermocouples, affecting applicator efficiency and the shape of the power deposition pattern. The applicators tested included the 434 MHz Beta applicator (Medlogix, Rome, Italy) connected to the 434 MHz ALBA ON 4000 device (Medlogix, Rome, Italy) [20,25], and the 915 MHz MA-100 applicator (Pyrexar, Salt Lake City, UT, USA) [26,27,28] connected to a 915 MHz generator (Elrad S.a.s., Milan, Italy). These applicators were used since they have comparable EFS and the TMS covers the entire applicator for clinical thermal monitoring. The main objective of the phantom tests is to evaluate whether the presence of the TMS affects applicator performance, including EFS, at both 434 and 915 MHz. 

#### 2.3.1. Applicator Efficiency

**Figure 3:**
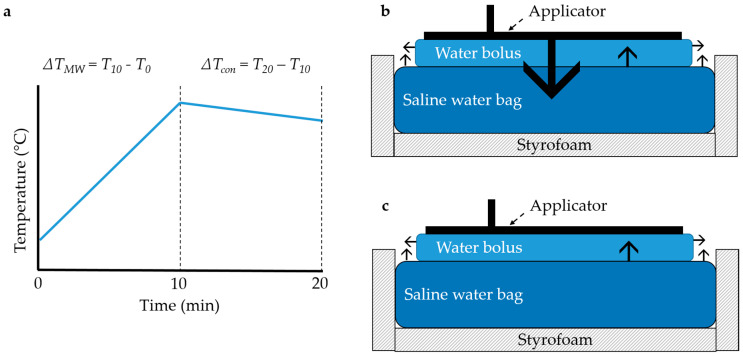
The experimental set-up to determine the applicator efficiency. (**a**) Power was applied for 10 min to the saline water bag which causes a temperature increase (Δ*T_MW_*), after turning off power the temperature decline due to conductive heat transport was measured for 10 min (Δ*T_con_*) to arrive at an estimate for the heat loss to the environment during heating (Equation (2)). (**b**) The heat transport during the heat up phase and (**c**) cool down phase is depicted with black arrows.

The influence of the TMS on the efficiency of the applicator to heat up a saline water bag (0.6% NaCl, dimensions: 350 × 250 × 90 mm) was investigated with the calorimetric method for both the Beta and MA-100 applicator (Figure 3). The applicators (room temperature, without water circulation) were placed on the saline water bag insulated with >20 mm Styrofoam on all sides except the top. The saline solution weighed 6.426 kg. Clinically relevant power of circa 50 W was given for 10 min, followed by a cool down (power off) period of 10 min. Continuous stirring of the saline solution was done with a magnetic stirrer (EM 3300 T, LaboTech, Zwolle, The Netherlands). The temperature of the saline solution was measured with a calibrated resistive temperature device (Pt100, temperature resolution 0.001 °C, accuracy ≤0.02 °C; Model 935-14-61, Isotech, Southport, UK). A calibrated power meter (U2001B, Keysight technologies, Santa Rosa, CA, USA) measured the power into the applicator via a bidirectional coupler with attenuation 20.52 and 15.62 dB for 434 and 915 MHz, respectively (attenuation determined with R&S ZNL Vector Network Analyzer, Rohde and Schwarz, Utrecht, The Netherlands). The efficiency (*η*) was determined by the ratio of energy uptake in the water bag to the energy delivered by the RF system:

(1) $$\eta =\frac{m\cdot C\cdot \Delta T}{P\cdot \Delta t}$$

where *P* (W) was the applied power, Δ*t* (seconds) the time of power on, *m* (kg) the mass of the saline solution, *C* (J/(kg °C)) the specific heat of water, and Δ*T* (°C) the temperature increase in 10 min calculated by:

∆*T* = ∆*T_MW_* − ½ ∙ ∆*T_con_* (2)

where Δ*T_MW_* (°C) was the measured temperature rise in the first 10 min, and Δ*T_con_* (°C) the temperature decrease due to environmental cool down in the second 10 min. The experiments were started at room temperature. Therefore, we assumed that the environmental cool down during the heat up phase was 50% of the environmental cool down during the cool down phase.

The experiment was repeated three times for each of the following cases in random order: applicator alone, applicator with TMS, and applicator with 10 µm high density polyethylene plastic foil. The mean efficiency and standard deviation for all scenarios were reported.

#### 2.3.2. Power Deposition Pattern

The influence of the TMS on the power deposition pattern of the Beta and MA-100 applicators was determined both with an IR camera and with an E-field probe.

**Figure 4:**
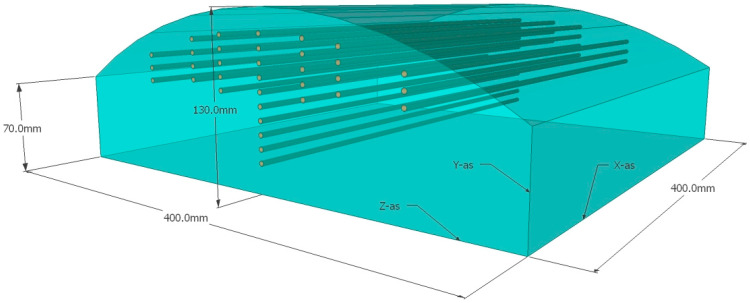
The slightly curved muscle-equivalent phantom used in the experiment to evaluate the power distribution pattern with the infrared (IR) camera. Catheters are positioned inside the phantom for invasive thermometry. The 10 mm thick fat-equivalent layer that can be positioned on top is not depicted. IR images are taken of the surface of the muscle-equivalent phantom and the 10 mm fat-equivalent layer.

The perturbation of the power deposition pattern on the surface and at 10 mm depth was investigated with an infrared camera (PI400/450, Optris GmbH, Berlin, Germany). A slightly curved muscle-equivalent phantom (dimensions (l × w × h): 400 × 400 × 70–130 mm) with on top a removable 10 mm thick fat-equivalent layer was used (muscle-equivalent and fat-equivalent at 434 MHz; Figure 4). The phantom was at room temperature and circa 50 Watt was applied for 10 min with the Beta and MA-100 applicators with and without the TMS. Before power on and immediately after turning power off an infrared photograph was made to determine the temperature rise of the surface of the fat-equivalent layer and, after removal of the layer, the surface of the muscle-equivalent phantom. The initial temperature rise reflects the power deposition pattern, i.e., the specific absorbed rate (SAR), for homogeneous nonperfused phantoms [13,29]. Changes in the width and length of the shape of the SAR distribution pattern were qualitatively evaluated, as well as variations in absolute temperature rise.

**Figure 5:**
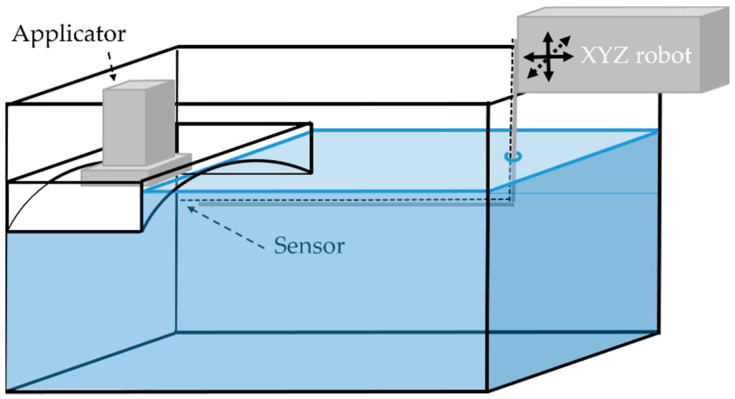
E-field probe measurements of the power deposition patterns were performed in a 1000 × 400 × 400 mm Plexiglas tank, filled with 80 L muscle-equivalent saline solution (0.6% NaCl). To mount the applicator in the tank, a Plexiglas mounting box with similar curvature as the phantom in Figure 4 was submerged in the larger Plexiglas tank. The E-field was automatically scanned using an E-field sensor (20 mm in-house built diode dipole with high resistance leads) attached to an in-house built positioning system (resolution 0.1 mm; XYZ robot assembled from parts from Igus, Cologne, Germany) in a 2.9 × 2.0 mm (X × Y) grid at 10 mm depth.

The influence of the TMS on the power deposition pattern was also investigated with E-field probe measurements in a 1000 × 400 × 400 mm Plexiglas tank, filled with 80 L muscle-equivalent saline solution (0.6% NaCl). The conductivity of the muscle-equivalent phantom was between 1.26 and 1.27 S m^−1^ (HD2106.2, Delta Ohm, Maarssen, The Netherlands). To mount the applicator in the tank, a Plexiglas mounting box with similar curvature as the phantom in Figure 4 was submerged in the larger Plexiglas tank (Figure 5). The E-field was automatically scanned using an E-field sensor (20 mm in-house built diode dipole with high resistance leads) attached to an in-house built positioning system (resolution 0.1 mm; XYZ robot assembled from parts from Igus, Cologne, Germany) in a 2.9 × 2.0 mm (X × Y) grid at 10 mm depth, where each step takes approximately 5 s. The E-fields of both the Beta and MA-100 applicator, while emitting circa 50 W, were scanned with and without the TMS. The EFS of the applicators for the different situations were determined. 

#### 2.3.3. Heat Transfer of the Water Bolus

**Figure 6:**
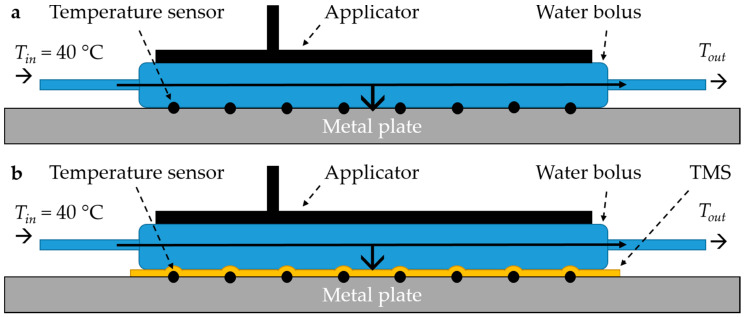
Experimental set-up to determine the heat transfer of (**a**) the water bolus alone, and (**b**) the water bolus with the thermal monitoring sheet (TMS).

The heat transfer coefficient *h* (W m^−2^ °C^−1^) from water to skin depends on the *thickness* (*m*) and thermal conductivity *k* (W m^−1^ °C^−1^) of the water bolus bag material [30]. The heat transfer coefficient of the water bolus may be affected by adding an additional layer of TMS material between the water bolus and the skin. During hyperthermia treatment, water is circulated through the water bolus with a certain temperature. Heat transport from the water bolus is directed to the patient (phantom), applicator and air. Experiments were performed to determine the heat transfer coefficient of the water bolus to the phantom (Figure 6). A one mm thick metal plate (440 × 280 mm) was used as a phantom and 8–10 seven-point copper-constantan thermocouple probes with 10 mm spacing were evenly distributed over the metal plate in a 130 × 130 mm and 170 × 180 mm rectangle to measure the surface temperature, for the MA-100 and Beta applicator, respectively. The applicators were placed on top of the rectangle with circulating water at circa 40 °C. To calculate the heat transfer coefficient the following formulas were used:

*h* = *q*/(*A* ∙ (*T_waterbolus_* − *T_surface_*)) (3)

where *q* (W) was the amount of transferred energy, *A* (m^2^) the surface area of the water bolus, *T_surface_* (°C) the average temperature of the surface of the phantom during 5 min of steady water temperature. *T_waterbolus_* (°C) was the average of the inflow and outflow of the water bolus during 5 min of steady state water temperature. The amount of transferred energy *q* (W), can be calculated by:

*q* = *S* ∙ *ν* ∙ *ρ* ∙ *C* ∙ (*T_in_ − T_out_*) (4)

where *S* ∙ *ν* was the volume flow rate (m^3^ s^−1^), *ρ* (kg m^−3^) the density of water, *C* (J kg^−1^ °C^−1^) the specific heat of water, and *T_in_ − T_out_* (°C) the temperature difference between the inflow and outflow of the water bolus. The volume flow rate of the water was measured at the inflow (FLM20-12PCW, Eggs DELTA, OVAL corporation, Japan). After achieving stable temperatures, the *T_in_*, *T_out_*, and *T_surface_* were determined as the median during a 5 min interval.

Each measurement was repeated three times and in random order. We compared the applicators alone (Figure 6a), applicators with TMS (Figure 6b), and the applicators with a 10 µm high density polyethylene plastic foil as presently used during superficial hyperthermia. The amount of transferred energy and the heat transfer coefficient for all scenarios were reported. We assumed that the heat transport to air and applicator was similar for all scenarios, therefore we did not establish these contributions since we wanted to focus on the effect of the TMS on heat transport between water bolus and patient skin.

### 2.4. Clinical Feasibility Study

Clinical feasibility of the TMS was evaluated in 10 patients with locoregional recurrent breast cancer in previously irradiated area treated with re-irradiation and hyperthermia at the Amsterdam University Medical Centers in May to August 2020. We investigated the effect of application of the TMS on treatment delivery regarding temperature monitoring of the skin surface and the effect of the TMS on thermal toxicity. All subjects gave their informed consent for inclusion before they participated in the study. The study was conducted in accordance with the Declaration of Helsinki, and the protocol was approved by the Ethics Committee of the Amsterdam University Medical Centers on March 31st 2020 (W20_111 # 20.146). Patients were re-irradiated to the chest wall with 32 Gy in 8 fractions of 4 Gy, two-weekly combined with weekly hyperthermia sessions. Hyperthermia treatment objectives were to elevate invasively measured target area temperatures to a minimum of 41 °C for 1 h while maintaining maximum surface temperatures below 43.5 °C. Hyperthermia started within 30–60 min after re-irradiation. Bendable conformal contact flexible microstrip applicators (Istok, Fryazino, Russia; Medlogix, Rome, Italy) operating at 434 MHz connected to the ALBA 4000 Double ON (Medlogix, Rome, Italy) were used for microwave heating (Table 2). The integrated water bolus containing temperature-controlled circulating deionized water was positioned between antenna and skin. Water temperature was adjusted to maintain a therapeutic temperature level of 42 °C on the skin surface.

Skin surface monitoring of the four consecutive hyperthermia treatments was done alternatingly with the TMS and standard thermometry methods of the Amsterdam UMC. A repeated measures design was used with six different sequences (SSTT, STST, STTS, TTSS, TSTS, TSST), where every patient received two treatments with the TMS (T) and two treatments with the standard thermometry method (S). The probes were placed perpendicular to the main direction of the EM field to avoid self-heating of the thermocouple probes. Power of the microwave device was on for 25 s and off for 5 s, to enable undisturbed temperature measurements. The clinical study started with the robust initial version of the TMS with a thickness of 0.5 mm, which was replaced during the study by the final, more flexible version with a thickness of 0.3 mm.

We investigated the effect of the TMS on the quality of treatment, compared to the standard thermometry method. The variation in invasive and skin surface temperatures, and applied power and water bolus temperature were evaluated. Furthermore, we investigated the duration to place the thermometry for the TMS vs. the standard method, the clinical usability, thermal toxicity, and hyperthermia related treatment complaints.

#### Statistical Analysis

The following treatment characteristics were evaluated for the treatments with TMS and the standard thermometry method. Hyperthermia treatment parameters were as follows: achieved invasive and skin temperature, median applied power and median water bolus temperature during steady state. The achieved temperature distribution was characterized using the T0 (maximum), T10, T50, T90, and T100 (minimum) and the difference between T0 and T100. The T10, T50, and T90 being the temperatures exceeded by 10%, 50%, and 90% of all measurement points during the steady state of the hyperthermia treatment, respectively. Differences between the TMS and the standard method in terms of these treatment characteristics were tested using linear mixed models (LMM) for repeated measures. Generalized linear mixed models (GLMM) were applied to analyze the effect of the TMS on thermal toxicity (blisters) and on the number of treatments with power related complaints (pain, heat, itching). Treatment characteristics may vary between patients and treatment sessions, therefore we used in all mixed-effect models a random intercept for patient and random slope for treatment sessions. Parameter estimates and 95% Wald confidence intervals (CI) were reported for fixed effects. If the 95% CI included zero, the results of models were not presented. We used R version 3.3.1 (R Foundation, Vienna) with package lme4 version 1.1-21 to perform the statistical tests and to estimate the parameters in the mixed-effect models.

## 3. Results

### 3.1. Phantom Experiments

#### 3.1.1. Applicator Efficiency

The mean efficiency ± standard deviation (SD) of the Beta applicator was 94.4% ± 2.9%. The TMS and plastic foil both decreased the absorbed power in the water bag, with 1.7% and 3.2%, respectively. The MA-100 applicator had a mean efficiency of 89.4% ± 1.5%. The applicator efficiency to heat up the saline water bag was reduced by 1.4% and 1.0% for the TMS and plastic foil, respectively (Table 3). 

#### 3.1.2. Power Deposition Pattern 

**Figure 7:**
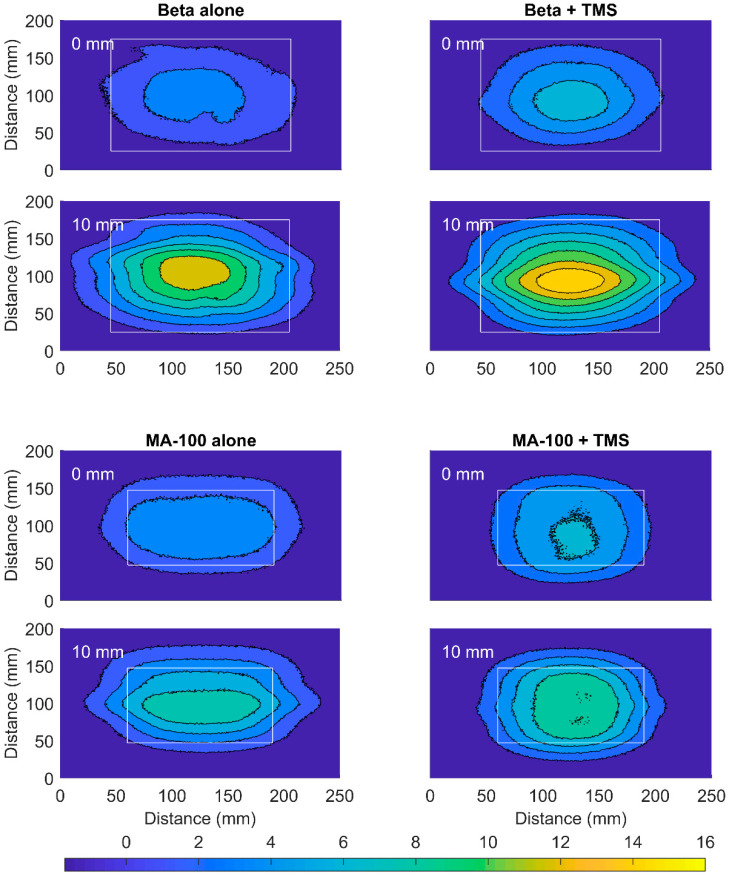
The infrared temperature data of the power deposition pattern of the Beta (51.0 W at 434 MHz; Medlogix, Rome, Italy) and MA-100 (44.1 W at 915 MHz; Pyrexar, Salt Lake City, USA) applicators. The temperature rise on the surface of the fat-equivalent layer is shown (0 mm) and at 10 mm depth on the surface of the muscle-equivalent phantom (10 mm). Results are displayed for the applicator alone (left) and the applicator combined with the thermal monitoring sheet (TMS; right). The aperture dimensions are represented by the white rectangle.

The TMS only slightly altered the power deposition pattern on the surface and at 10 mm depth measured with an IR camera, for both 434 and 915 MHz (Figure 7). The applied power was 51.0 and 44.1 W for the Beta and MA-100 applicator, respectively. Placement of the TMS between applicator and phantom increased the temperature of the surface of the fat-equivalent layer (maximum increase MA-100: 0.3 °C; Beta: 2.3 °C) and, at 10 mm depth, of the surface of the muscle-equivalent layer (maximum increase MA-100: 0.7 °C; Beta: 2.1 °C). Minor changes in the width and length of the shape of the power distribution pattern of both the Beta and MA-100 applicator were introduced by the TMS.

**Figure 8:**
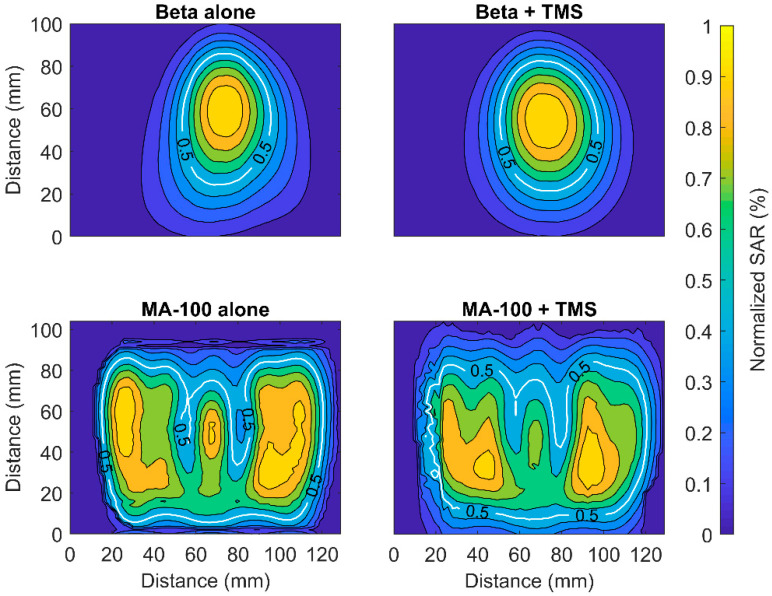
Normalized SAR distribution of the Beta and MA-100 applicators alone (left) and the applicators combined with the thermal monitoring sheet (TMS; right) at 10 mm depth in a liquid muscle-equivalent phantom. The effective field of the applicators is depicted in white.

Furthermore, the normalized SAR (E^2^) measured with an E-field probe was also only slightly affected by the placement of the TMS (Figure 8). The measurements for 434 MHz were done with power of 50.0 W with phantom that had σ of 1.265 S m^−1^, respectively. The 915 MHz applicator emitted 45.8 W, the phantom had σ of 1.261 S m^−1^, respectively.

The EFS of the Beta applicator was 43 × 58 mm, and increased slightly with the TMS, to 47 × 58 mm. The MA-100 applicator alone had EFS 80 × 105 mm and decreased slightly by the TMS to 76 × 105 mm (Table 3).

#### 3.1.3. Heat Transfer of the Water Bolus

**Figure 9:**
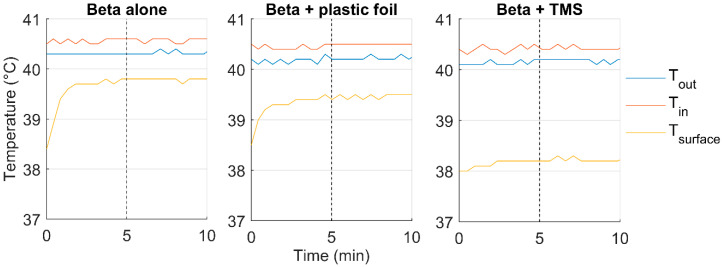
The effect of the 10 µm high density polyethylene plastic foil and thermal monitoring sheet (TMS) on the heat transfer of the water bolus of the Beta applicator. The 5 min steady state interval starts after the dotted vertical line. The median temperature of the inflow (*T_in_*), outflow (*T_out_*), and the median surface temperature (*T_surface_*) are presented during a 10 min interval.

The TMS and plastic foil did reduce the heat transfer to the phantom for both applicators (Table 3). For the Beta applicator, the heat transfer coefficient was 2288 ± 123 W m^−2^ °C^−1^, this was reduced by 56.4% and 26.6% due to the presence of the TMS and plastic foil, respectively. For the MA-100 applicator, the heat transfer coefficient was 2717 ± 406 W m^−2^ °C^−1^. The TMS reduced the heat transfer coefficient by 50.5% of the original value, while the plastic foil reduced it by 11.1%. Volume flow rate during the experiments was ≈1.2 L min^−1^. Figure 9 displays the measured temperatures during one of the heat transfer experiments for each of the three scenarios.

### 3.2. Clinical Study

**Figure 10:**
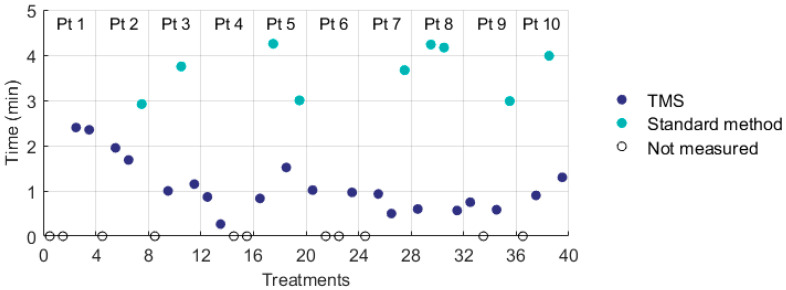
The duration to position skin surface temperature sensors with the thermal monitoring sheet (TMS) and with the Amsterdam UMC standard method for thermometry.

Ten women were included for the study, who received in total 20 treatments with the TMS and 20 treatments with the Amsterdam UMC standard method for skin surface thermometry. All patients had surgically removed locoregional recurrent breast cancer, five patients had mastectomy scars and five patients had lattisimus dorsi (LD) reconstructions, of whom one patient had a silicon prosthesis in situ. Due to the dimensions of the target region, best coverage could be achieved with the 3H applicator (Istok, Fryazino, Russia) and all patients were treated with this applicator, which is the largest and most used applicator in our department. The effective field size of the 3H applicator (177 × 175 mm) was adequately covered by the monitoring area of the TMS (162.5 × 140 mm). The treatment characteristics were presented in Table 4. The invasive temperature beneath the 3H applicator was measured in nine patients during 32 treatments. For the TMS and the standard method, both the invasive and skin temperatures (T0, T10, T50, and T90) were not significantly different. Analysis using linear mixed models showed that the minimum skin temperature decreased significantly with 0.8 °C when using the TMS (95% CI −1.5 to −0.1 °C). The difference between maximum and minimum skin temperature was significantly higher with TMS (1.0 °C; 95% CI 0.1 to 1.8 °C). The time needed to position the temperature sensors was measured during 29/40 treatments and significantly decreased from ≈3.5 min to ≈1 min by using the TMS (Table 4; Figure 10). 

Neither method resulted in any thermal toxicity (i.e., blisters). Treatments with the TMS appeared to result in slightly more hyperthermia treatment related complaints (i.e., pain, hot, itching) than treatments with the standard method, although this was not statistically significant. In one patient with a LD reconstruction the TMS was used during the first treatment and a hotspot occurred on the lower left side of the LD reconstruction, a location where standardly no thermometry would be positioned. This hotspot was seen during all consecutive treatments. It is likely that since the hotspot was noticed by application of the TMS, the occurrence of thermal toxicity was prevented in this patient. The TMS was user friendly and easy to clean.

The 0.3 mm TMS was applied during nine treatments in five patients, in all other treatments the 0.5 mm TMS was used. Comparing the results of both TMS did not result in significant differences in power, water bolus temperature, or achieved temperature. The 0.5 mm TMS resulted in four treatments with complaints, the 0.3 mm TMS in two treatments with complaints.

## 4. Discussion 

The influence of the TMS on the antenna performance was investigated in phantom experiments and in a clinical feasibility study. The phantom experiments showed that application of the TMS did neither significantly affect the shape of the power deposition, nor the applicator efficiency (from −1% to −2%). The heat transfer of the water bolus was reduced by ca. 50% due to the presence of the TMS (Table 3), whereas the standard thermometry using a 10 µm foil as applied at Amsterdam UMC reduced the heat transfer by 11–27%. A decrease in the cooling ability of the water bolus might induce a higher risk of thermal toxicity. The maximum temperature and thermal dose on the skin surface are prognostic for thermal toxicity [4,7,31,32,33,34,35]. The decreased heat transfer can be compensated by increasing the volume flow rate through the water bolus.

In the clinical feasibility study, no thermal toxicity occurred using the TMS. The skin surface temperature distribution was safely and adequately monitored with the TMS during superficial hyperthermia treatments. The decrease in heat transfer of the water bolus resulted in more heterogeneity of the skin surface temperature; the variation between the maximum and minimum surface temperatures was 1.0 °C (0.1–1.8 °C) higher for treatments with the TMS. The application of the TMS instead of the Amsterdam UMC standard of care thermometry, reduced the set-up time of hyperthermia treatment with approximately 2.5 min. This slightly reduced the 30–60 min time-interval between radiotherapy and hyperthermia treatment, further reduction requires faster transport of the patient between radiotherapy and hyperthermia, or reversing the sequence to first hyperthermia followed by radiotherapy [36]. A shorter time interval is associated with better treatment outcome both in animal studies and in cervical cancer patients, so this might also further improve response to treatment of patients with locoregional recurrent breast cancer [37,38].

The TMS provides high resolution monitoring of the skin surface temperature, which is correlated to the occurrence of thermal toxicity in patients with locoregional recurrent breast cancer [4,7]. Accurate measurement of the temperature distribution of the skin surface is important to prevent the occurrence of thermal toxicity. However, the achieved skin surface temperature has not been proven to correlate with the tumor response, local control, and overall survival in patients with locoregional recurrent breast cancer in a previously irradiated area [4]. To improve treatment outcome, it remains necessary to also monitor invasive temperatures. 

Although clinical feasibility was demonstrated, the design of the TMS could be further improved. For typical chest wall recurrences, the surface is sufficiently regular for adequate positioning of the TMS, but the TMS is not applicable to patients with a highly irregular surface of the target area. Although the TMS is flexible and slightly stretchable, in these patients too much air would be present between the target surface area and the applicator to allow adequate coupling of the water bolus [15]. For these patients the skin surface temperatures can be measured by placement of individual temperature probes. The presence of thermocouple probes in the TMS requires placement of the TMS such that the probes are aligned perpendicular to the main direction of the EM field. Thus, adequate temperature measurements using the TMS can only be guaranteed when combined with applicators that have one dominant E-field direction. These metal probes also require that the power of the microwave hyperthermia device has to be switched off in repeated intervals for 5 s to allow accurate temperature measurement. The use of fiber-optic probes or high resistance thermistors in a TMS would overcome this and allow the TMS to be placed in any direction, while the microwave hyperthermia device can continuously emit power.

The TMS will be implemented as the standard of care for skin surface temperature monitoring during hyperthermia treatments performed with equipment operating at 434 MHz at the Amsterdam University Medical Centers. This high-resolution temperature monitoring, using 56 rather than eight or less sensors is potentially able to reduce the incidence of thermal toxicity during superficial hyperthermia treatment [9], and thereby to improve treatment quality. Specifically, if the sensors are placed in a reproducible manner and include critical structures such as scar tissue. Improvement in treatment quality by the TMS should be confirmed in a large-scale, prospective, comparative study in hyperthermia centers that standardly use eight temperature sensors for thermal monitoring. During alternating treatments, their standard of care thermometry should be compared with thermometry by the TMS for each participating patient, similar to the procedure followed in the present study. Another advantage of using a TMS is the presentation of the temperature distribution of the skin surface on a 2-D grid, which allows the operator to evaluate the presence and localization of any cool or hotspots on the treatment surface. The implementation of the TMS allows easier comparison of treatment data of different sessions of a single patient, or sessions between patients, and even makes interinstitutional comparison possible.

## 5. Conclusions

The application of a high-resolution thermal monitoring sheet was suitable for clinical microwave superficial hyperthermia. Phantom experiments showed that the TMS did not significantly alter the power deposition patterns and applicator efficiency of both 434 and 915 MHz applicators, though the TMS reduced the heat transfer of the water bolus.

A clinical feasibility study in 10 patients strongly suggests that it is feasible to monitor the skin surface temperatures with the TMS without enhancing thermal toxicity. The TMS had no adverse effects. The TMS can be implemented for thermal monitoring of the skin surface during superficial hyperthermia treatment.

## Figures and Tables

**Table 1:** Specifications of the applicators used in the phantom experiments and the clinical study; the Beta applicator (Medlogix, Rome, Italy), MA-100 applicator (Pyrexar, Salt Lake City, USA), and 3H applicator (Istok, Fryazino, Russia). SAR: specific absorbed rate.

Specifications	Phantom Experiments		Clinical Study
	Beta Applicator	MA-100 Applicator	3H Applicator
Frequency	434 MHz	915 MHz	434 MHz
Applicator (main direction EM-field ↕)	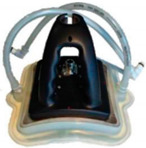	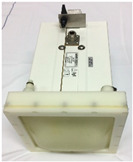	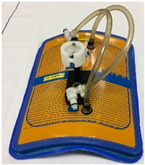
Aperture dimensions	150 × 160 mm [17]	100 × 130 mm [18]	290 × 210 mm [19]
Aperture shape	Fixed curvature	Flat	Bendable
Water bolus dimensions	170 × 175 mm	130 × 130 mm	300 × 220 mm
Effective field size (50% SAR)	80 × 120 mm [17]	80 × 100 mm [18]	177 × 175 mm [20]
Effective heating depth (25% SAR)	23–33 mm [17]	25 mm [18]	30 mm [19]

**Table 2:** Overview of the phantom experiments.

Experiment	Method	Phantom	Comparison
Applicator efficiency	Calorimetric method (Figure 3)	Insulated saline water bag Dimensions: 350 × 250 × 90 mm (l × w × h) Content: 6.426 kg; 0.6% NaCl	Applicator Applicator with TMS Applicator with standard of care: 10 µm high density polyethylene plastic foil
Power deposition pattern	IR camera (Figure 4)	Curved muscle-equivalent phantom with on top a removable 10 mm fat-equivalent layer Muscle-equivalent phantom: Dimensions: 400 × 400 × 70–130 (top) mm Content: wallpaper paste [21,22,23] Fat-equivalent layer: Dimensions: 400 × 400 × 10–70 (top) mm Content: acetylene black mixed with aluminum powder and laminac polyester resin [24]	Applicator Applicator with TMS
	E-field (Figure 5)	Tank filled with liquid muscle-equivalent phantom with a curved top Dimensions: 1000 × 400 × 400 mm Content: ca. 80 L; 0.6% NaCl; σ = 1.26–1.27 S m^−1^	Applicator Applicator with TMS
Heat transfer of the water bolus	(Figure 6)	Metal plate Dimensions: 440 × 280 × 1 mm	Applicator Applicator with TMS Applicator with standard of care: 10 µm high density polyethylene plastic foil

**Table 3:** Results of the phantom experiments for applicator efficiency, power distribution pattern (effective field size), and of the heat transfer of the water bolus. Results are presented for the applicators alone, applicators with a 10 µm high density polyethylene plastic foil, or applicators with the thermal monitoring sheet (TMS) positioned between the water bolus and the phantom.

Parameter	Quantity (Unit)	Beta Applicator			MA-100 Applicator		
		Alone	+Plastic Foil	+TMS	Alone	+Plastic Foil	+TMS
Applicator efficiency (%)	Mean ± SD	94.4 ± 2.9	91.2 ± 2.1	92.7 ± 1.1	89.4 ± 1.5	88.5 ± 1.7	88.0 ± 1.5
	Reduction (%)		3.2	1.7		1.0	1.4
Power distribution pattern	Effective field size E-field probe (mm)	43 × 58		47 × 58	80 × 105		76 × 105
Heat transfer coefficient *h* (W m^−2^ °C^−1^)	Mean ± SD	2288 ± 123	1680 ± 160	997 ± 32	2717 ± 406	2417 ± 199	1345 ± 241
	Reduction (%)		26.6	56.4		11.1	50.5

**Table 4:** Treatment characteristics of 10 patients for treatments with the standard skin surface thermometry method of the Amsterdam University Medical Centers compared to the thermal monitoring sheet (TMS). The results of the (generalized) linear mixed models were presented.

Treatment Characteristic		Standard Method	TMS	(Generalized) Linear Mixed Models	
				Parameter Estimate	95% CI
Treatments		20	20		
Power (W)		44.5 ± 11.4	48.4 ± 12.3		
Water bolus temperature (°C)		42.5 ± 0.6	42.6 ± 0.7		
Invasive temperature (°C) ^1^	T0	41.6 ± 1.1	42.0 ± 1.5		
	T10	41.0 ± 0.9	41.3 ± 1.3		
	T50	39.8 ± 1.1	39.8 ± 1.3		
	T90	38.8 ± 1.0	38.8 ± 1.1		
	T100	38.1 ± 0.6	38.1 ± 0.9		
	T0 − 100	3.5 ± 1.0	3.9 ± 1.5		
Skin temperature (°C)	T0	43.2 ± 0.4	43.4 ± 0.5		
	T10	41.9 ± 0.3	41.9 ± 0.2		
	T50	40.9 ± 0.3	40.8 ± 0.3		
	T90	39.8 ± 0.3	39.6 ± 0.5		
	T100	38.4 ± 0.9	37.3 ± 1.4	−0.8	−1.5 to −0.1
	T0 − T100	4.8 ± 1.0	5.7 ± 1.7	1.0	0.1 to 1.8
Time to place the sensors (s) ^2^		220 ± 34	66 ± 36	−159	−184 to −134
Treatments with thermal toxicity		0	0		
Treatments with complaints		3	6		

Abbreviations: T0, maximum temperature); T10, T50, and T90, the temperatures exceeded by 10%, 50%, and 90% of all measurement points during the steady state of the hyperthermia treatment; T100, minimum temperature. Results are presented with mean ± standard deviation. Parameter estimates and 95% Wald confidence intervals (CI) of (generalized) linear mixed models where the 95% CI did not include zero are presented. ^1^ Invasive temperature was measured in 9/10 patients. ^2^ The time to place the temperature sensors was measured during 29/40 treatments.
